# Multifractality of random eigenfunctions and generalization of Jarzynski equality

**DOI:** 10.1038/ncomms8010

**Published:** 2015-04-27

**Authors:** I.M. Khaymovich, J.V. Koski, O.-P. Saira, V.E. Kravtsov, J.P. Pekola

**Affiliations:** 1 Low Temperature Laboratory, Department of Applied Physics, Aalto University, FI-00076 Aalto, Finland; 2 Department for Physics of Superconductivity, Institute for Physics of Microstructures, Russian Academy of Sciences, 603950 Nizhny Novgorod, GSP-105, Russia; 3 Condensed Matter and Statistical Physics Section, Abdus Salam International Center for Theoretical Physics, Strada Costiera 11, 34151 Trieste, Italy; 4 L. D. Landau Institute for Theoretical Physics, 2 Kosygina street, 119334 Moscow, Russia

## Abstract

Systems driven out of equilibrium experience large fluctuations of the dissipated work. The same is true for wavefunction amplitudes in disordered systems close to the Anderson localization transition. In both cases, the probability distribution function is given by the large-deviation ansatz. Here we exploit the analogy between the statistics of work dissipated in a driven single-electron box and that of random multifractal wavefunction amplitudes, and uncover new relations that generalize the Jarzynski equality. We checked the new relations theoretically using the rate equations for sequential tunnelling of electrons and experimentally by measuring the dissipated work in a driven single-electron box and found a remarkable correspondence. The results represent an important universal feature of the work statistics in systems out of equilibrium and help to understand the nature of the symmetry of multifractal exponents in the theory of Anderson localization.

## Abstract (web summary)

The work fluctuations of systems driven out of equilibrium are governed by the same large-deviation theory as wavefunction amplitudes close to the Anderson localization transition. Exploiting this analogy, the authors generalize the Jarzynski equality, verifying their relation on a single-electron box.

## Graphical abstract

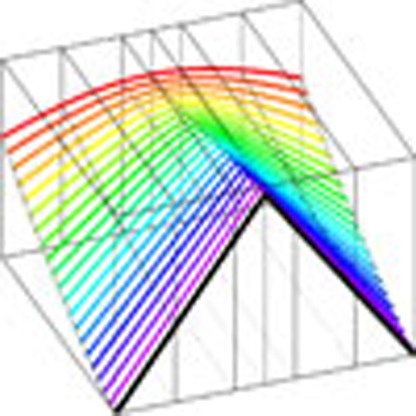

Unlike the adiabatic processes where the work *W* done on the system is equal to the difference in the free energy Δ*F*, the non-adiabatic drive protocols are associated with work that depends not only on the parameters of the system and details of the drive protocol but also experiences fluctuations relative to its average value^1^,^2^,^3^,^4^,^5^,^6^,^7^,^8^. Statistics of work can be described by the probability distribution function (PDF), *P*_*w*_(*W*), and it is an important goal to find universal features in *P*_*w*_(*W*) that remain unchanged within certain universality classes^9^. The best known relations of this kind are the Jarzynski equality^10^,^11^,^12^ and the Crooks relation^13^. The former one states that the exponent 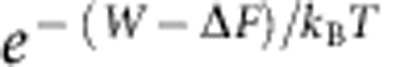 averaged over repeated identical driving protocols is equal to 1, where *T* is the temperature of the single bath and *k*_B_ is the Boltzmann constant. This necessarily implies that during some drive realizations the dissipated work *W* −Δ*F* must be negative in a naive (and wrong) contradiction with the second law, which only states that the average dissipated work remains positive. The Crooks relation


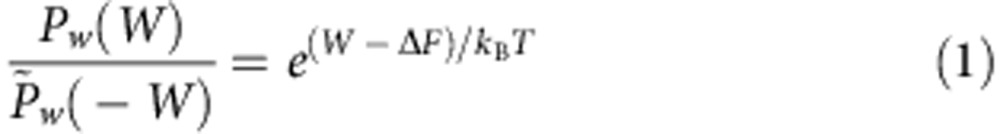


concerns the PDFs of work in the direct (*P*_*w*_(*W*)) and time-reversed (
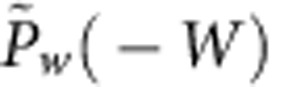
) processes. This relation has many important consequences (with the Jarzynski equality being one of them) and practical applications, for example, in the determination of free energy of folding proteins^1^,^5^.

We use the Crooks relation to find a correspondence between statistics of work in a broad class of systems driven by time-reversal symmetric protocols and statistics of random multifractal wavefunctions in disordered quantum systems close to the Anderson localization transition^14^,^15^,^16^. The unifying principle of this correspondence^17^ is the so-called large-deviation principle^18^ according to which the PDF of a large variety of systems takes the form of the large-deviation ansatz (LDA),


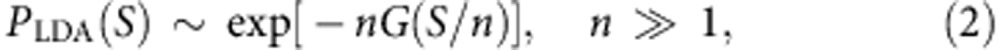


where *G*(*y*) is a system-specific function. The LDA can be viewed as a generalization of the Central Limit Theorem of statistics according to which the sum *S* of a large number *n* of identically distributed independently fluctuating quantities *s*_*k*_ has a limiting Gaussian distribution with the variance *σ*^2^∝*n*. Indeed, if we require that *G*(*y*) in equation (2) has a minimum, the expansion of this function near this minimum immediately results in the correct Gaussian PDF. The significance of the LDA is that it also describes the non-Gaussian tails of the distribution. Different realizations of LDA are characterized by different functions *G*(*y*) and different effective number *n* of independently fluctuating quantities. For example, in the discrete Markov process (or Markov chain) driving time *t* plays the role of large parameter *n* for steady-state distributions of dissipated work^19^ and heat^20^.

Critical eigenfunctions 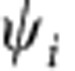 (*i*=1, ... *N*) near the Anderson localization transition and in certain random matrix ensembles have multifractal statistics^14^,^15^,^16^. A characteristic feature of such statistics is that the eigenfunction amplitude 
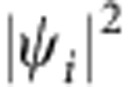
 takes a broad set of values (at different sites *i* or in different realizations of disorder) that scale like 
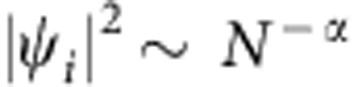
 (*α*>0) with the total number of sites *N* in a disordered tight-binding lattice (or the matrix size). The number of sites on a lattice where scaling is characterized by a certain *α* is *M*∼*N*^*f*(*α*)^ , where *f*(*α*) is known as the spectrum of fractal dimensions. Where *α* is taking only one single value *α*_0_, the set of ‘occupied' sites on the *d*-dimensional lattice would be a fractal with the Hausdorff dimension *d*_*h*_=*d·f*(*α*_0_). Multifractality implies that there is a range of possible values of *α* with the corresponding range of fractal dimensions *f*(*α*). In the language of LDA, this implies that PDF of the amplitude 
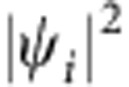
 has a form equation (2) with 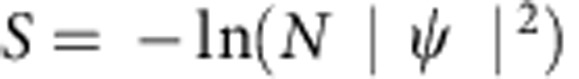 and *n*=ln *N*. The function *G*(*y*) is related with the multifractality spectrum *f*(*α*) as *G*(*y*)=1−*f*(1+*y*)^14^. It depends on parameters of the system such as the dimensionality or the bandwidth of the random matrix ensemble, and has a non-trivial limit at *N*→∞. There is a remarkable symmetry^14^,^17^,^21^,


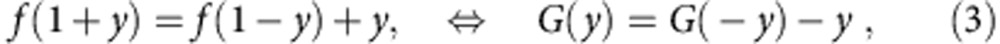


whose physical origin is perhaps deeper than a current understanding^22^ based on ‘full chaotization' of particle dynamics in a random potential, which leads to the homogeneous distribution of the scattering phase off the disordered system.

An important observation^17^ with potentially very far-reaching consequences is that within the LDA the symmetry; equation (3) is equivalent to the Crooks-like relation,


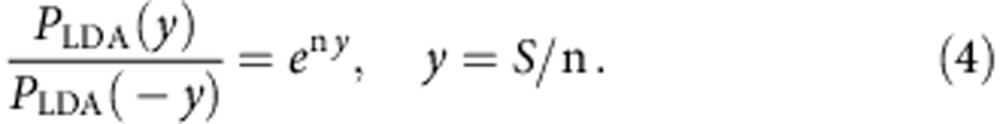


In this work, we formulate a generalization, equation (7), of the Jarzynski equality for the work-generating function. This generalization has been proven theoretically by a stochastic calculus using the rate equations and experimentally for a driven single-electron box (SEB) in the Coulomb blockade regime.

## Results

### The large-deviation parameter and the temperature

To formulate a dictionary between the statistics of work in driven systems and that of random multifractal eigenfunctions, we compare equations (1) and (4) assuming that the drive protocol in equation (1) is time-reversal symmetric, therefore 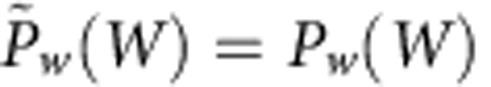. Using this comparison and the definition of *S* and *n* for multifractal wavefunctions, we obtain


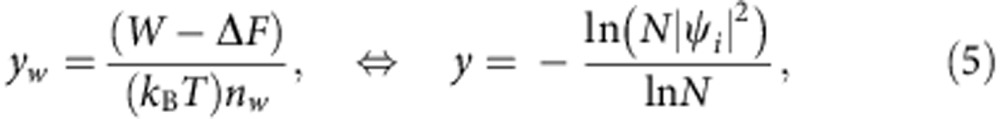


where the subscript *w* stands for the distribution of work fluctuations. To determine the yet undefined parameter *n*_*w*_, we use the following heuristic argument based on the above analogy. We note that for a normalized eigenfunction on a lattice obeying 
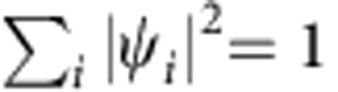
 we have 
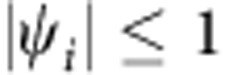
. This means that *y*≥−1. A similar restriction for *y*_*w*_ implies (*W*−Δ*F*)/*k*_B_*T* ≥ − n_*w*_, that is


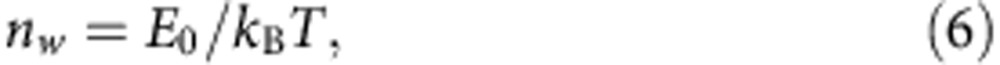


where (−*E*_0_) is the lower bound of the dissipated work. This result for the large-deviation parameter *n*_*w*_ can be proven by a usual stochastic approach (see equation (15) in the Supplementary Note 1) for the SEB governed by rate equations obeying detailed balance. However, we believe that it is valid generically for all driven systems with the dissipated work bounded from below. Thus the effective number (equation (6)) of independent random variables in such driven systems is inversely proportional to temperature *T* and is easily controllable experimentally. This result is crucially important for experimental verification of our extension of the Jarzynski equality.

### Work-generating function and extension of the Jarzynski equality

**Figure 1:**
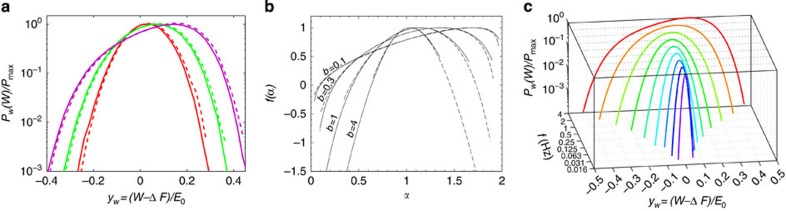
Comparison of distributions of dissipated work and amplitudes of random multifractal wavefunctions. (**a**) Distribution of the measured normalized dissipated work (*W*−Δ*F*)/*E*_0_ on the logarithmic scale. The width of the distributions increases with increasing drive frequency *f*=1 (red), 2 (green) and 4 Hz (violet) at temperature *T*=214 mK. (**b**) Multifractality spectrum *f*(*α*) of critical eigenfunctions in disordered systems close to the Anderson localization transition versus normalized logarithm of wavefunction intensity 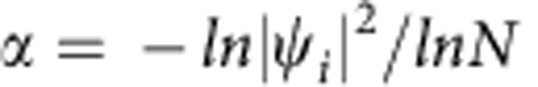 for the power-law random banded matrix model with the bandwidth *b*=0.1, 0.3, 1, 4 (adapted with permission from ref. 21. Copyrighted by the American Physical Society). This parameter is known to mimic the dimensionality of space in which the Anderson transition happens: *b*→0 corresponds to the limit of infinite dimensionality *d*→∞, or the Bethe lattice limit, while *b*→∞ corresponds to *d*=2+*ɛ*, where *ɛ* →+0. In both **a** and **b**, solid and dashed lines correspond to *G*(*y*), *G*(−*y*)−*y* and *f*(*α*), *f*(2−*α*)+*α*−1, respectively, to demonstrate the symmetry (equation (3)). (**c**) Evolution of distribution of the normalized dissipated work (*W*−Δ*F*)/*E*_0_ on the logarithmic scale with decreasing drive frequency *f* in a SEB with a superconducting external electrode for experimental system parameters and *T*=214 mK obtained theoretically from the rate equations (equations (3) and (4) in the Supplementary Note 1). The width of the distributions decreases with decreasing driving frequency *f* (from red to violet curve). The similar calculations for the SEB with the normal electrode give 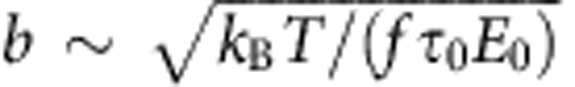, where *τ*_0_ is the characteristic relaxation time of the circuit. Thus an effective bandwidth *b* of the equivalent random matrix theory depends on the equivalent size of the matrix *N*=e*xp*(*E*_0_/*k*_B_*T*). While the limit *T*→0 always corresponds to the limit *b*→0, the limit *f*→0 at a fixed *T* corresponds to the adiabatic limit *b*→∞.

**Figure 2:**
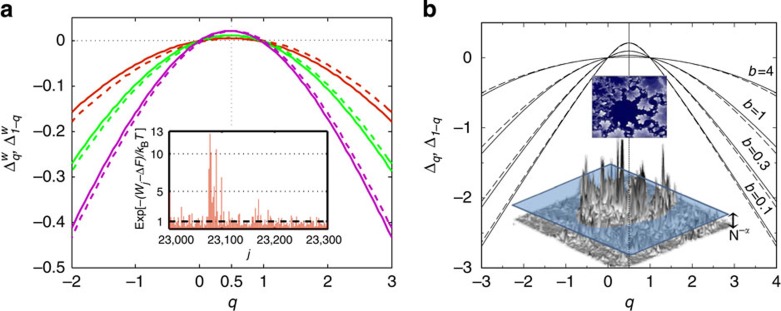
Comparison of 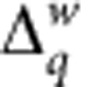 for dissipated work and multifractal critical exponents Δ_*q*_. (**a**) The measured function 
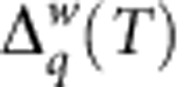
 in equation (7) at drive frequencies *f*=1 (red), 2 (green) and 4 Hz (violet) and temperature *T*=214 mK. (Inset) A plot of the exponent of the dissipative work 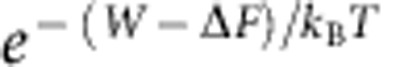 versus the drive realization number *j* at a drive frequency *f*=1 Hz and temperature *T*=214 mK. In most of the drives the exponent is smaller than 1, which corresponds to *W*>Δ*F*, as the second law requires for averages. However, there are rare events seen as high spikes when Δ*F*−*W*>*k*_B_*T*. (**b**) Multifractal exponents Δ_*q*_ for the same model and parameters as in Fig. 1b (adapted with permission from ref. 21. Copyrighted by the American Physical Society). In both **a** and **b** small difference between Δ_*q*_ (solid lines) and Δ_1 − *q*_ (dashed lines) violating the symmetry (equation (8)) is due to experimental (in **a**) or numerical (in **b**) errors. (Bottom inset) A plot of a typical amplitude 
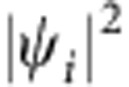
 of the critical wavefunction in a two-dimensional lattice with *N* sites cut at a certain level 
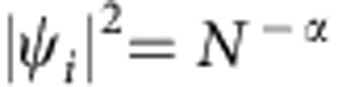
 (adapted with permission from ref. 39. Copyrighted by the American Physical Society). (Top inset) The map of the region in space where 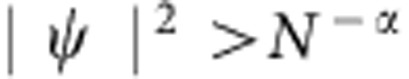 is a fractal of the Haussdorf dimension *d*_h_(*α*)=2*f*(*α*)<2. Multifractality implies a dependence of *d*_h_ on *α*, or on the cutoff level *N*^−*α*^ (adapted with permission from ref. 40. Copyrighted by the American Physical Society).

With the established physical meaning of *n*_*w*_, the analogy between the work distribution in driven systems and the multifractal statistics of random eigenfunctions becomes complete. It is illustrated in Figs 1 and 2.

A remarkable property of the LDA (equation (2)) is that the average of 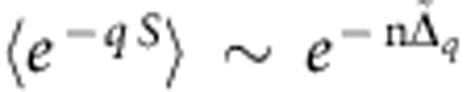 is an exponential function of *n*≫1, where 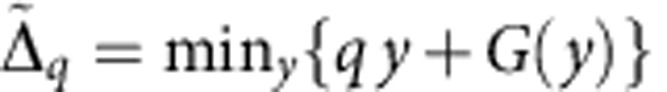
18,19. Given that *n*=ln*N*, this implies a power-law scaling with *N* of the moments 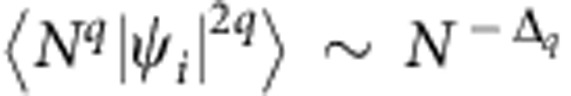 (with 
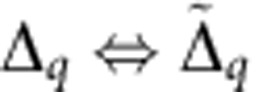
) of random wavefunctions near the critical point of the Anderson localization transition. When applied to the statistics of work, the exponential dependence on *n*_*w*_=*E*_0_/*k*_B_*T* results in the following relation for the work-generating function 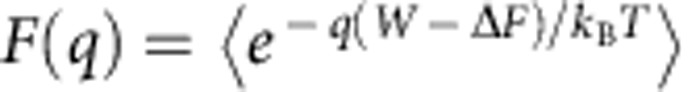:


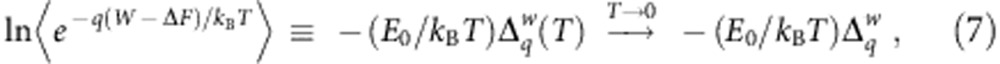


where the limit 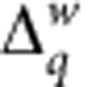 is independent of temperature. Equation (7) is the main theoretical result of our work, where we claim that the logarithm of the work-generating function is linear in *E*_0_/*k*_B_*T*≫1, with 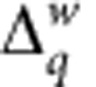 being a non-trivial function of a real *q*. It generalizes the Jarzynski equality, which corresponds to *q*=1 and 
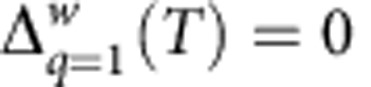
. Apparently, we have also 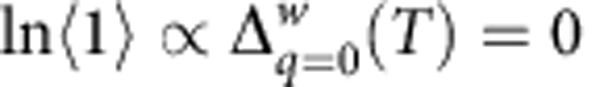. One can easily show using equation (3) and the definition of 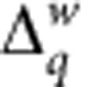 that the symmetry


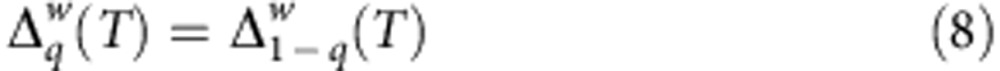


holds both for 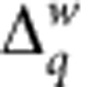 and for 
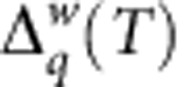
. This symmetry has its counterpart for the critical exponents Δ_*q*_ that determine the scaling with *N* of the moments of random critical wavefunctions. The limit 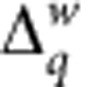 of 
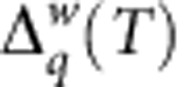
 at *E*_0_/*k*_B_*T*→∞ (at a fixed drive frequency *f*) is expected to be robust to changing the details of the driven system and the drive protocol. For a driven two-level system, described by rate equations (equations (3) and (4)) in the Supplementary Note 1) and obeying detailed balance 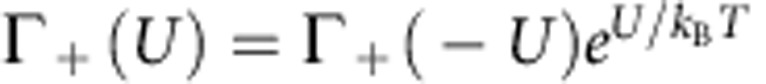 for the up (down) transition rates Γ_+_ (Γ_−_), standard stochastic dynamics calculus confirms the main result equations (6) and (7), with 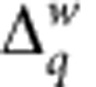 having always the same asymptotic behaviour 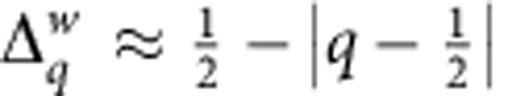 at large enough |*q*|≫*q*_c_ (see equation (18) in the Supplementary Note 1). This form of 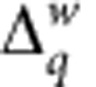 corresponds to the limit of infinite dimensions, or the Bethe lattice limit^23^, in the problem of the random critical wavefunctions. Note that the universal behaviour of 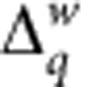 (and the corresponding behaviour of *P*_*w*_(*W*)) is reached only in the limit *T*→0, with all other parameters of the system and drive being fixed. If, however, the temperature is low but fixed, then there always exists a sufficiently low drive frequency *f* such that the dissipated work distribution tends to a *δ*-function, as the adiabatic limit requires^24^. For a SEB with a superconducting electrode, the range of such frequencies could be extremely low at temperatures *k*_B_*T*<<Δ_S_, with Δ_S_ being a superconducting gap in the island (see Fig. 1c).

### Experimental verification for a SEB

**Figure 3:**
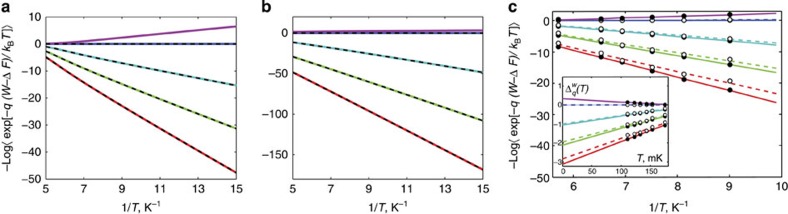
The dependence in *T*^−1^ of the logarithm of the work-generating function and its symmetry in the moment order *q*. In **a** and **b**, the theoretical *T*^−1^ dependence obtained from the rate equations for a SEB with (**a**) a superconducting and (**b**) a normal external electrode is shown. (**c**) Demonstration of the experimental test of this dependence. In all panels the dependencies become linear at large values of *T*^−1^. The dashed (solid) lines correspond to the pairs of moments {*q*,1−*q*} related by symmetry. The curves from bottom to top correspond to {4, −3}, {3, −2}, {2, −1}, {1,0} and 
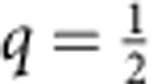
. In **c**, the dashed (solid) lines of the same colour are linear fits of the experimental data shown by open (solid) circles corresponding to *q* (1−*q*). (Inset) The linear in *T* extrapolation for the function 
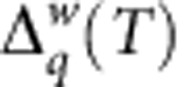
. The notations are the same as in **c**.

**Figure 4:**
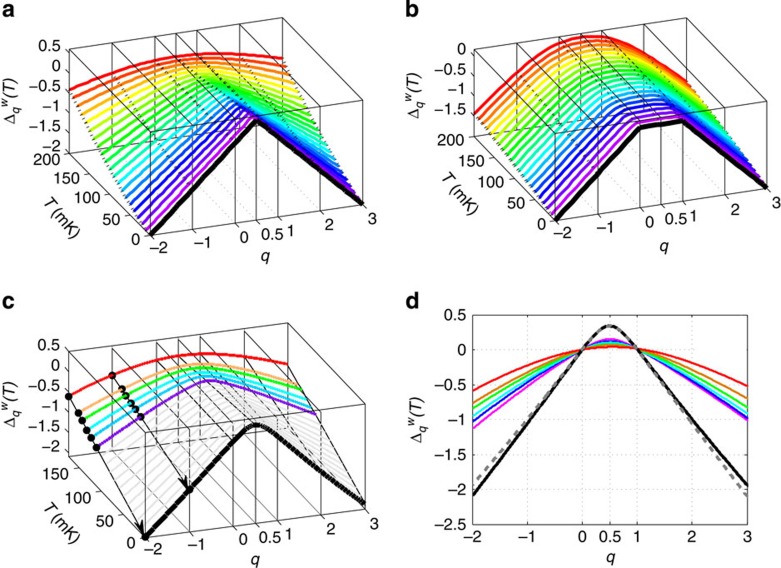
Evolution of 
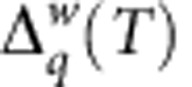
 with decreasing temperature *T* in a single-electron box. (**a**,**b**) The theoretical evolution for a SEB with (**a**) a superconducting external electrode for Δ_S_=*E*_C_ and (**b**) a normal external electrode obtained from the rate equations. The limiting 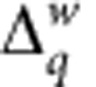 is of (**a**) triangular, (**b**) trapezoidal form. Experimental data are shown in **c** and **d**. (**c**) Experimentally obtained 
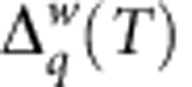
 for temperatures *T*=111, 123, 133, 144, 153 and 175 mK are plotted as functions of *q* (coloured solid lines). The solid black curve 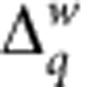 is obtained by linear in *T* extrapolation of the experimental data to zero temperature. The thin grey curves and dotted arrows demonstrate the linear extrapolation. (**d**) The verification of the symmetry (equation (8)). Here the dashed grey curve shows 
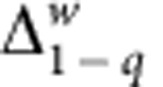
, the other notations are the same as in **c**.

The general theory above can be applied to a driven SEB, which is a small metallic island connected to an external electrode with a tunnel junction. The free electrons on the SEB island and the electrode form a particle bath, assumed to be at thermal equilibrium at temperature *T* (refs 6, 25). A standard rate equation approach^26^,^27^, which is essentially classical and based on the picture of sequential tunnelling of electrons, confirms our main result (equation (7)) and the symmetry (equation (8)) (see Supplementary Notes 1 and 2). This theory gives a linear in *T*^−1^ low-temperature behaviour of the cumulant generating function (left-hand side of equation (7)), as shown in Fig. 3a,b. We consider two different cases as examples belonging to different universality classes marked by a drastically different dependence of the tunnelling rate Γ_+_(*U*) on the drive voltage *U*≫*k*_B_*T*: a SEB with normal island and (a) a superconducting external electrode (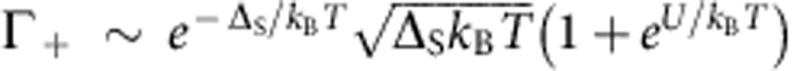, Δ_S_>*U*) or (b) a normal external electrode (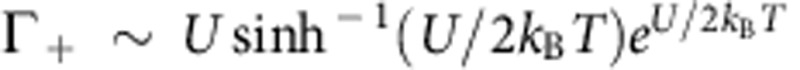). The evolution of 
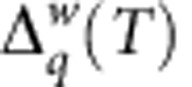
 with temperature in both cases is shown in Fig. 4. The limiting 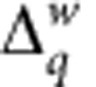 appears to be of triangular shape in case (a), and of trapezoidal shape in case (b).

The main quantum effects, which are beyond the rate equation approach, are the elastic co-tunnelling^28^ and the Andreev tunnelling^29^. Estimations show (see equation (3) and the Supplementary Note 3) that for SEB at our experimental conditions, they may become relevant at low temperatures *T*<T*∼60 mK. We believe that these effects merely renormalize the parameter *E*_0_ and the function 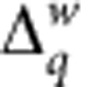 and do not change the 1/*T* behaviour in equation (7). Further investigations are necessary to check the validity of this conjecture.

For an experimental verification of our main result (equation (7)) and the symmetry (equation (8)), we use a SEB formed by two metallic islands, of which one is normal and the other one is superconducting with energy gap Δ_S_. As a two-island SEB is only capacitively coupled to the environment, it is less influenced by external noise from higher temperature stages of the set-up. Otherwise its behaviour is identical to a normal one-island SEB with a superconducting ‘external electrode'. The measured structure is described in refs 6, 30, 31. We used aluminium and copper as a superconductor and a normal metal, respectively, and apply magnetic field to increase the tunnelling rates through the junction by suppressing the gap Δ_S_, see the Supplementary Note 4 for details. The Hamiltonian *H*(*n*,*n*_g_)=*E*_C_(*n*^2^−2*n n*_g_) of the SEB consists of the charging energy of the island with an integer number of excess electrons *n* and the interaction with the source of the gate voltage *V*_g_ controlling the gate charge *n*_g_=*C*_g_*V*_g_/*e* through the capacitance *C*_g_. The energy required to charge the island with a single electron −*e* is *E*_C_=*e*^2^/2*C*_Σ_, where *C*_Σ_ is the total capacitance of the island. In this experiment, we apply a sinusoidal modulation 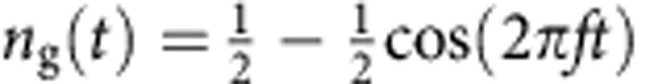 and consider a monotonous segment of *n*_g_(*t*) from 0 to 1 as a single realization of the process 0<*t*<(2*f*)^−1^. We focus on the large Coulomb energy limit *E*_C_≫*k*_B_*T*, in which *n* is restricted to two values, *n*=0 and *n*=1. In this case, the dissipated work is determined from the trajectory of *n*(*t*) by^24^,^32^


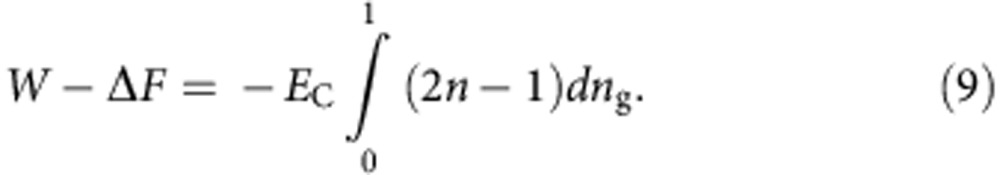


Like in the textbook example of a moving piston where the volume *V*(*t*) of the gas is controlled deterministically and the pressure *p*(*t*) experiences fluctuations due to collisions of gas atoms with the piston, the gate voltage *n*_g_(*t*) is a deterministic function, whereas *n*(*t*) experiences telegraph fluctuations. These fluctuations are detected by a nearby charge-sensitive single-electron transistor. The dissipated work is computed from equation (9) and its statistics over repeated identical driving protocols is studied. Here the lower bound −*E*_0_ of the dissipated work is determined by the Coulomb energy *E*_0_=*E*_C_.

The experimental PDFs of work for few different drive frequencies are presented in Fig. 1. This plot demonstrates the dependence of the PDF on the frequency, which is reminiscent of the dependence on the bandwidth *b* of the corresponding PDF for random multifractal wavefunctions for the power-law banded random matrices^14^. Using this PDF one can compute the *q*th moments of 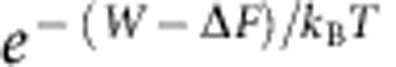 for different values of the parameter *q* and find the function 
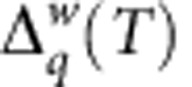
 from equation (7) (see Fig. 2). In both figures the charging energy of the SEB determining the dissipated work equation (9) is *E*_C_=167±4 μeV, while the bath temperature is *T*=214 mK. The drive frequencies are indicated in the figures.

Next, we check experimentally the linear in *E*_0_/*k*_B_*T* low-temperature dependence in equation (7) and the symmetry of equation (8). The results are presented in Fig. 3c. The corresponding theoretical curves are given in Fig. 3a,b. Note a good linearity of experimental data for the negative *q* (full circles, solid lines) and a much larger scatter of it (open circles) for the large positive *q*, which corresponds to rare events with *W*−Δ*F*<0. The linear in *T* evolution of 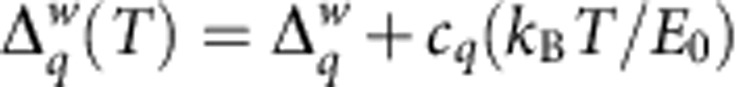 is demonstrated experimentally in Fig. 4c. Its counterpart for the random eigenfunction problem is the evolution with the system size *N* linear in 1/ln*N*, which was used recently in ref. 23 to find the spectrum of fractal dimensions *f*(*α*) extrapolated to the infinite system size. Similarly to this work, the limiting function 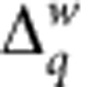 is obtained by the linear in *T* extrapolation to *T*→0 (see the inset in Fig. 3c). In both figures, the charging energy is *E*_C_=111±4 μeV, the drive frequency is *f*=4 Hz, while the temperatures are indicated in the figures. The estimated^33^,^34^,^35^ value of the superconducting energy gap Δ_S_=96±11 μeV in applied magnetic field is rather close to *E*_C_ in this case. The corresponding extrapolated function 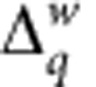 shown in Fig. 4c is close to the triangular form obtained theoretically from the rate equations in the ideal case Δ_S_=*E*_C_ and shown in Fig. 4a, albeit it is somewhat rounded on the top following a trend towards the trapezoidal form shown in Fig. 4b. The asymptotic behaviour of the extrapolated function 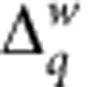 at *q*>1 or *q*<0 is close to the theoretically predicted asymptotics 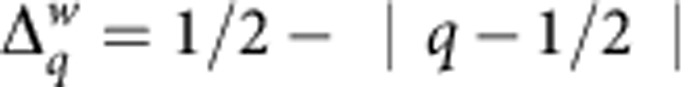, linear in *q* with unit slope, supporting the linear in *T* extrapolation.

## Discussion

In conclusion, we have shown that the analogy between the statistics of random critical wavefunctions and that of the work dissipated in driven systems is very suggestive. Its predictions are fully confirmed theoretically and experimentally by studying stochastic dynamics in a driven SEB described by rate equations obeying detailed balance. Thus one of the most difficult problems in quantum mechanics of disordered systems turns out to be analogous to one of the simplest problem in classical stochastic Markovian dynamics. In particular, the physical origin of the symmetry (equation (8)) is somewhat unclear in the problem of Anderson localization (but see ref. 22). At the same time, the corresponding symmetry for driven systems is a consequence of the Crooks relation or, equivalently, of detailed balance for rate equations. This might suggest that there would be a stochastic description for the critical random eigenfunction problem by an equivalent Markovian process with detailed balance. One can see a remote analogy of such correspondence in the Schramm–Loewner evolution, which maps fractal phase boundaries in two-dimensional critical systems onto a simple random walk on a line^36^,^37^,^38^.

## Author contributions

J.V.K., O.-P.S. and J.P.P. conceived and designed the experiments. J.V.K. and O.-P.S. performed the experiments. I.M.K., J.V.K. and O.-P.S. analysed the data. I.M.K., V.E.K. and J.P.P. contributed with materials/analysis tools. I.M.K., J.V.K., V.E.K. and J.P.P. wrote the paper.

## Additional information

**How to cite this article:** Khaymovich, I. M. *et al.* Multifractality of random eigenfunctions and generalization of Jarzynski equality. *Nat. Commun.* 6:7010 doi: 10.1038/ncomms8010 (2015).

## Supplementary Material

Supplementary Information Supplementary Notes 1-4 and Supplementary References
