# Supplementary material for: Multifractality of random eigenfunctions and generalization of Jarzynski equality
Source: Nat Commun. 2015 Apr 27;6:7010. doi: 10.1038/ncomms8010 (PMC4421851; doi:10.1038/ncomms8010)
Supplement: Supplementary Information — Supplementary Notes 1-4 and Supplementary References [file ncomms8010-s1.pdf]

I. SUPPLEMENTARY NOTE 1 STATISTICS OF WORK FOR A SINGLE-ELECTRON BOX

In this part we give a sketch of the calculation of PDF $P_w(W_{\rm d})$ of dissipated work $W_{\rm d} = W - \Delta F$ in a SEB which confirms Eq. (7) from the main text and the asymptotics $\Delta_q^w = 1/2 - |q-1/2|$ for a simple Markovian system described by rate equations. For the sake of clarity we consider the time-reversal (anti)symmetric protocol of the gate voltage $n_{\rm g}(t)$ monotonously increasing from 0 to 1 in time $0 < t < \tau = (2f)^{-1}$, i.e., $n_{\rm g}(\tau - t) = 1 - n_{\rm g}(t)$, where the symmetry (1) is satisfied with $\tilde{P}_w(W) = P_w(W)$. As was mentioned in the main text we also focus on the large Coulomb energy limit $E_{\rm C} = e^2/[2(C_{\rm g} + C)] \gg k_{\rm B}T$. Then the excess number of electrons n on the island is restricted to two values $n = 0$ and $n = 1$ in this range of gate voltage, $0 \leq n_{\rm g}(t) \leq 1$.

The Hamiltonian $H(n, n_{\rm g}) = E_{\rm C}(n^2 - 2n_{\rm g}n)$ of a SEB mentioned in the main text determines the dissipated work as follows[1]:

$$W_{\rm d}[n(t), n_{\rm g}(t)] = W - \Delta F = -E_{\rm C}\int_0^1 (2n-1)dn_{\rm g} \ , \tag{1}$$

with the (thermodynamical) work $W = \int \frac{\partial H}{\partial n_{\rm g}} dn_{\rm g}$, and the free energy difference $\Delta F = F(1) - F(0)$, where $\beta F(n_{\rm g}) = -\ln\left[\sum_n e^{-\beta H(n, n_{\rm g})}\right]$ and $\beta = (k_{\rm B}T)^{-1}$. For the chosen drive protocol the minimal dissipated work $-E_0 = \min W_{\rm d} = -E_{\rm C}$ is negative with the absolute value coinciding with the Coulomb energy.

During the ramp of $n_{\rm g}(t)$ different stochastic trajectories $n(t)$ of the charge state occur with alternating jumps of n either from 0 to 1 or vice versa. Each trajectory unambiguously determines the dissipated work for a realization, see Eq. (1). To find the distribution of dissipated work

$$P_w(W_{\rm d}) = \sum_{k=0}^{\infty}\sum_{\eta=0}^{1}\int P_k(\eta, t_1, \ldots, t_k)\delta\left(W_{\rm d}[n(t), n_{\rm g}(t)] - W_{\rm d}\right) dt_1 \cdot \ldots \cdot dt_k \tag{2}$$

one calculates the probability $P_k(\eta, t_1, \ldots, t_k)$ of realizing a trajectory $n(t)$ starting at $n(0) = \eta = \overline{0, 1}$ which has k jumps occuring at time instants $t_1, \ldots, t_k$. For this purpose we solve the master equation for the occupation probabilities p_n of the two charge states

$$\frac{dp_1}{dt} = \Gamma_+(t)p_0 - \Gamma_-(t)p_1, \quad p_0 = 1 - p_1 \ , \tag{3}$$

with the equilibrium initial state $p_1(0) = \Gamma_+(0)/[\Gamma_+(0) + \Gamma_-(0)] \approx e^{-\beta E_C}$. Here the rates $\Gamma_\pm(t)$ of electron tunneling into/out of the island can be written as follows $\Gamma_\pm(t) = \Gamma[\pm U(t)]$ with the function

$$\Gamma[U] = \frac{1}{e^2 R_{\rm T}}\int_{-\infty}^{\infty} \nu(E) f_T(E)[1 - f_T(E+U)]dE \ , \tag{4}$$

monotonically increasing with $U(t) = H(0, n_{\rm g}) - H(1, n_{\rm g})$:

$$U(t) = E_{\rm C}[2n_{\rm g}(t) - 1]. \tag{5}$$

Here $R_{\rm T}$ is the tunnel resistance of the contact and we consider a normal-metal island with the constant density of states, while the external electrode can be either in a normal (N) or in a superconducting (S) state with the normalized density of states equal to $\nu(E) = 1$ or $\nu(E) = \left|{\rm Re}\frac{E}{\sqrt{E^2 - \Delta^2}}\right|$, respectively. We also assume that electrons in the island and in the electrode thermalize quickly enough to have the same Fermi distributions of energy $f_T(E) = \left(e^{\beta E} + 1\right)^{-1}$ with the temperature T of the single bath. One of the main consequences of the latter assumption about thermalization and Eq. (4) is the detailed balance of the tunneling rates

$$\Gamma_+(t)/\Gamma_-(t) = e^{\beta U(t)} \ , \tag{6}$$

which eventually results in the Crooks relation (1) for dissipated work (1) in the system described by rate equation[2,3] and driven by time-reversal (anti)symmetric drive

$$U(\tau - t) = -U(t), \ \texttt{when} \ \ \Gamma_+(\tau - t) = \Gamma_-(t) \ . \tag{7}$$

In the low temperature limit $k_{\rm B}T/E_{\rm C}\to 0$ (at a fixed drive frequency $f=(2\tau)^{-1}$) we can restrict our consideration to zero- and one-jump trajectories starting from the ground state $n(0)=0$. The probabilities of considered trajectories can be calculated in a similar way as in Ref. 4

$$P_0(\eta) = p_\eta(0)e^{-A} , \tag{8}$$
$$P_1(0,t_1) = p_0(0)e^{-I(t_1)}\Gamma_+(t_1) , \tag{9}$$

where $A=\int_0^\tau \Gamma_+(t)dt$ and $I(t_1)=\int_0^{t_1}\Gamma_+(t)dt+\int_{t_1}^\tau \Gamma_-(t)dt$ is symmetric, $I(\tau-t)=I(t)$, due to (7), it is bounded $0\le I\le A$ and $I(0)=A$. The validity of considering only zero- and one-jump trajectories can be verified using the following estimate for the total probability of trajectories with $k>1$ jumps,

$$P_{k>1}=1-\sum_{\eta=\pm}P_0(\eta)-\int_0^\tau P_1(0,t_1)dt_1\le I(\tau/2) , \tag{10}$$

which vanishes in the limit $T\to 0$. Indeed,

$$\int_0^\tau P_1(0,t_1)dt_1 \approx \int_0^\tau e^{-I(t_1)}\Gamma_+(t_1)dt_1 =$$
$$\int_{U(t)>0} e^{-I(t_1)}\left[\Gamma_+(t_1)+\Gamma_-(t_1)\right]dt_1 \ge \int_{U(t)>0} e^{-I(t_1)}\dot{I}(t_1)dt_1 = e^{-I(\tau/2)}-e^{-A} , \tag{11}$$

and

$$P_{k>1}\le 1-e^{-I(\tau/2)}\le I(\tau/2) , \tag{12}$$

where

$$I(\tau/2)=2\int_{U(t)>0}\Gamma[U(t)]e^{-\beta U(t)}dt \lesssim \frac{\Gamma_0\tau}{\beta E_{\rm C} n_{\rm g}'(\tau/2)}\to 0 \tag{13}$$

and $n_{\rm g}'(\tau/2)=\partial n_{\rm g}/\partial(t/\tau)$ is the derivative of the gate voltage $n_{\rm g}$ over the normalized time. Here we used the symmetry (7) and the natural assumption that the maximal value of the tunneling rates $\Gamma[E_{\rm C}]$ is bounded to $\Gamma[E_{\rm C}]<\Gamma_0=\text{const}$ in the considered limit of $k_{\rm B}T/E_{\rm C}\to 0$.

As a result, Eq. (2) yields

$$P_w(y_w)\approx e^{-A}\left[\delta(y_w-1)+e^{-\beta E_{\rm C}}\delta(y_w+1)\right]+\frac{\tau}{2}\Gamma[E_{\rm C}\cdot y_w]e^{-I(y_w)} , \tag{14}$$

where y_w is defined in Eq. (5) of the main text.The singular part of P_w corresponds to the trivial jumpless trajectories. They make a contribution to $\Delta_q^w(T)-\Delta_q^w(0)=O(1/(\beta E_0))$ in Eq. (7) from the main text which is subleading. The regular part of $P_w(y_w)$ can be compared with the large deviation ansatz of Eq. (2) from the main text by taking the limit $G(y)=\lim_{\mathfrak{n}\to\infty}G(y,\mathfrak{n})$ of $G_w(y_w,\mathfrak{n}_w)\equiv -\ln[P_w(y_w)]/\mathfrak{n}_w$ as

$$G_w(y_w,\mathfrak{n}_w)=-\frac{\ln\gamma(y_w)}{\mathfrak{n}_w}+\frac{I(y_w)-\ln(\Gamma[E_{\rm C}]\tau/2)}{\mathfrak{n}_w} . \tag{15}$$

Here $\mathfrak{n}_w=\beta E_{\rm C}$, $\gamma(y_w)=\Gamma[E_{\rm C}y_w]/\Gamma[E_{\rm C}]$ and the second fraction vanishes when $T\to 0$. The first term gives the main contribution to $G_w(y_w)$ and for the normal external electrode $\gamma(y_w)=y_w(1-e^{-\beta E_{\rm C}y_w})^{-1}$ we obtain

$$G_w(y_w)=\begin{cases}-y_w, & -1\le y_w\le 0\\ 0, & 0\le y_w\le 1\end{cases} , \tag{16}$$

while for the superconducting external electrode with $\gamma(y_w)=e^{-\beta E_{\rm C}}(1+e^{\beta E_{\rm C}y_w})$ the function $G_w(y_w)$ takes the form

$$G_w(y_w)=\begin{cases}1, & -1\le y_w\le 0\\ 1-y_w, & 0\le y_w\le 1\end{cases} . \tag{17}$$

The corresponding limiting $\Delta_q^w = \min_{y_w} \{y_w\, q + G(y_w)\}$ is

$$\Delta_q^w \to \frac{1}{2} - \left| q - \frac{1}{2} \right| \ , \tag{18}$$

for the case of the superconducting external lead, while for the normal external lead the positive part of Δ_q^w in Eq. (18) at $0 < q < 1$ is replaced by 0. In both cases the asymptotic behavior $\Delta_q^w \approx \frac{1}{2} - \left|q - \frac{1}{2}\right|$ for $q < 0$ or $q > 1$ holds true.

In general for low finite temperatures, $k_{\rm B}T \ll E_{\rm C}$, the averaging in Eq. (7) from the main text can be calculated using the saddle-point approximation and we obtain

$$\Delta_q^w(T) = \min_{y_w} \{y_w\, q + G(y_w, \mathfrak{n}_w)\} = \Delta_q^w + c_q(T)/\mathfrak{n}_w \ . \tag{19}$$

Here the last term $c_q(T)/\mathfrak{n}_w$ originates from the second fraction in Eq. (15) with bounded $c_q(T) < c_q^{\max} = \text{const}$ and therefore it is a subleading term. As a result, rewriting Eq. (7) from the main text in the following form

$$\ln\langle e^{-q\,(W-\Delta F)/k_{\rm B}T}\rangle = -(E_0/k_{\rm B}T)\,\Delta_q^w + c_q(T) = -(E_0/k_{\rm B}T)\,\Delta_q^w + O(1) \ , \tag{20}$$

we prove the linear behavior of the l.h.s. in $E_0/k_{\rm B}T$.

The linear T-expansion of $\Delta_q^w(T)$, Eq. (19), to Δ_q^w is possible in the range of temperatures, where $c_q(T)$ is close to its limiting value $c_q(0)$, i.e., when $c_q(T) \approx c_q(0)$.

II. SUPPLEMENTARY NOTE 2 DERIVATION OF EQ. (8)

The symmetry $\Delta_q^w(T) = \Delta_{1-q}^w(T)$ of Eq. (8) can be proved simply using Crooks relation for time-reversal symmetric drive protocol (4): $P_w(y_w) = e^{\mathfrak{n}_w y_w} P_w(-y_w)$. Indeed,

$$e^{-\mathfrak{n}_w \Delta_q^w(T)} = \int e^{-q\mathfrak{n}_w y_w} P_w(y_w) dy_w = \int e^{(1-q)\mathfrak{n}_w y_w} P_w(-y_w) dy_w = \int e^{-(1-q)\mathfrak{n}_w y_w'} P_w(y_w') dy_w' = e^{-\mathfrak{n}_w \Delta_{1-q}^w(T)} \ . \tag{21}$$

III. SUPPLEMENTARY NOTE 3 ESTIMATION OF THE COHERENT PROCESSES IN SIN SEB

In this section we estimate temperature range where coherent phenomena, namely, Andreev tunneling[5], start to affect dynamics of single-electron box (see Eq. (3) in Supplementary Note 1). For this purpose we compare the minimal rate of sequential tunneling (see Eq. (4) in Supplementary Note 1 for $U = -E_{\rm C}$) with typical amplitudes of Andreev tunneling rates. Strictly speaking there is another coherent effect called cotunneling, but it is not relevant to a SEB dynamics due to one tunnel junction in the system.

Substituting the expression for the superconducting density of states $\nu(E) = \left|{\rm Re}\frac{E}{\sqrt{E^2-\Delta_{\rm S}^2}}\right|$ into Eq. (4) in Supplementary Note 1 one can find the expression for the sequential tunneling rate at $k_{\rm B}T \ll U < \Delta_{\rm S}$:

$$\Gamma[U] \approx \frac{\sqrt{2\pi\Delta_{\rm S} k_{\rm B}T}}{e^2 R_{\rm T}} e^{-\Delta_{\rm S}/k_{\rm B}T}\left(1 + e^{U/k_{\rm B}T}\right) \tag{22}$$

with the minimal value $\Gamma[-E_{\rm C}] \simeq \sqrt{2\pi\Delta_{\rm S} k_{\rm B}T} e^{-\Delta_{\rm S}/k_{\rm B}T}/e^2 R_{\rm T}$ at $E_{\rm C} < \Delta_{\rm S}$.

In subgap region $|U| < \Delta_{\rm S}$ Andreev tunneling rate $\Gamma_{\rm AR}$ can be estimated as follows:

$$\Gamma_{\rm AR} = \frac{R_{\rm Q}}{8e^2 N R_{\rm T}^2}|U| \ , \tag{23}$$

where $R_{\rm Q} = h/e^2 = 26$ kΩ is the resistance quantum, N is the the effective number of channels in the contact.

Using the experimental parameters $R_{\rm T} \sim 12$ GΩ, $|U| \sim \Delta_{\rm S} \sim E_{\rm C} \sim 100$ μeV, $N \sim 100$ we obtain the following threshold temperature T^* by comparing the rates $\Gamma[-E_{\rm C}] \sim \Gamma_{\rm AR}$

$$T^* \sim \Delta_{\rm S}/k_{\rm B} \ln[8R_{\rm T}N/R_{\rm Q}] \sim 1.15\ {\rm K}/\ln[4\cdot 10^8] = 60\ {\rm mK} \ , \tag{24}$$

below which the coherent effects cannot be neglected. This estimation agrees very well with the experimental observation of individual Andreev events in a hybrid single-electron transistors.[6]

IV. SUPPLEMENTARY NOTE 4 ENERGY GAP IN THE SUPERCONDUCTOR SUPPRESSED BY MAGNETIC FIELD

Experimentally we increase the tunneling rates $\Gamma[U]$ through the NIS junction in the measurements shown in Figs. 3(c) and 4(c) in the main text by applying magnetic field to control the energy gap Δ_S in the quasiparticle spectrum. Here we estimate the energy gap $\Delta_S(H)$ near the junction under the influence of the magnetic field $H = 475$ G applied to the superconducting island made of aluminium. We focus only on the energy gap value near the junction because the tunneling rates are governed by the local density of states of the superconductor near the junction, as given by Eq. (4) in Supplementary Note 1.

Based on a typical normal-state resistivity $\rho_N = 30 - 40$ nΩ·m of aluminum at 4.2 K,[7,8] we estimate the diffusion coefficient $D = 70 - 90$ cm^{2}s^{-1} from the Drude formula $1/\rho_N = e^2 N_0 D$, where $N_0 = 1.45 \cdot 10^{47}$ J^{-1}m^{-3} is the normal state density of states of aluminium.[9] According to this estimate the elastic mean free path $\ell \sim 10$ nm is small-compared to the superconducting coherence length $\xi_0 = \sqrt{\hbar D/\Delta_S(0)} \sim 140 - 165$ nm corresponding to the superconducting gap in aluminium at zero magnetic field $\Delta_S(0) \simeq 220$ µeV. The width of the island near the junction is estimated to be $w = 100$ nm using scanning electron microscope image of the sample.

Due to the inequalities $\ell \ll w \lesssim \xi_0$, one can assume homogeneous suppression of the gap

$$\Delta_S(H) = \Delta_{OP}(H)(1 - \gamma_H^{2/3})^{3/2} , \tag{25}$$

where the expression $\Delta_{OP}(H) = \Delta_S(0)\left(1 - 0.75\gamma_H - 0.54\gamma_H^2\right)$ for the superconducting order parameter in the magnetic field holds for $\gamma_H \lesssim 0.3$. These expressions follow from the solution to the Usadel[10] or Gor'kov[11,12] equations. Note that in zero magnetic field the order parameter coincides with the energy gap $\Delta_{OP}(0) = \Delta_S(0)$. Here, $\gamma_H = \frac{1}{6}\left(\frac{H\xi_0 w}{\hbar/e}\right)^2$. This results in the estimate $\Delta_S(H) = 96 \pm 11$ µeV for the gap in the presence of the magnetic field applied in the present experiment, which is the value used in the main text, and which agrees with the measured temperature dependence of the tunneling rates (not shown).

1. Pekola, J. P. and Saira, O-P. Work, free energy and dissipation in voltage driven single-electron transitions. *J. Low Temp. Phys.* **169,** 70-76 (2012).
2. Crooks, G. E. Entropy production fluctuation theorem and the nonequilibrium work relation for free energy differences. *Phys. Rev. E* **60,** 2721-2726 (1999).
3. Monthus, C., Berche, B. and Chatelain, C. Symmetry relations for multifractal spectra at random critical points. *J. Stat. Mech.* P12002 (2009).
4. Averin, D. V. and Pekola, J. P. Statistics of the dissipated energy in driven single-electron transitions. *Europhys. Lett.* **96,** 67004 (2011).
5. Averin, D. V. and Pekola, J. P. Nonadiabatic Charge Pumping in a Hybrid Single-Electron Transistor. *Phys. Rev. Lett.* **101,** 066801-1 - 066801-4 (2008).
6. Maisi, V. F., Saira, O.-P., Pashkin, Yu. A., Tsai, J. S., Averin, D. V., and Pekola, J. P. Real-Time Observation of Discrete Andreev Tunneling Events. *Phys. Rev. Lett.* **106,** 217003-1 - 217003-4 (2011).
7. Timofeev, A. V., Helle, M., Meschke, M., Möttönen, M. and Pekola, J. P. Electronic Refrigeration at the Quantum Limit. *Phys. Rev. Lett.* **102,** 200801 (2009).
8. Peltonen, J. T., Virtanen, P., Meschke, M. Koski, J. V. Heikkilä, T. T. and Pekola, J. P. Thermal Conductance by the Inverse Proximity Effect in a Superconductor. *Phys. Rev. Lett.* **105,** 097004 (2010).
9. Knowles, H. S., Maisi, V. F. and Pekola, J. P. Probing quasiparticle excitations in a hybrid single electron transistor. *Appl. Phys. Lett.* **100,** 262601 (2012).
10. Anthore, A., Pothier, H. and Esteve, D. Density of States in a Superconductor Carrying a Supercurrent. *Phys. Rev. Lett.* **90,** 127001 (2003).
11. Skalski, S., Betbeder-Matibet, O. and Weiss, P. R. Properties of Superconducting Alloys Containing Paramagnetic Impurities. *Phys. Rev.* **136,** A1500-A1518 (1964).
12. Maki, K. and Fulde, P. Equivalence of Different Pair-Breaking Mechanisms in Superonductors. *Phys. Rev.* **140,** A1586-A1592 (1965).
